# Environmental and Intrinsic Correlates of Stress in Free-Ranging Wolves

**DOI:** 10.1371/journal.pone.0137378

**Published:** 2015-09-23

**Authors:** Barbara Molnar, Julien Fattebert, Rupert Palme, Paolo Ciucci, Bruno Betschart, Douglas W. Smith, Peter-Allan Diehl

**Affiliations:** 1 Institute of Biology, University of Neuchâtel, Neuchâtel, Switzerland; 2 School of Life Sciences, University of KwaZulu-Natal, Durban, South Africa; 3 Department of Biomedical Sciences/Unit of Physiology, Pathophysiology and Experimental Endocrinology, Vetmeduni, Vienna, Austria; 4 Department of Biology and Biotechnologies, University “La Sapienza”, Roma, Italy; 5 Yellowstone Center of Resources, National Park Service, Yellowstone National Park, Wyoming, United States of America; Michigan Technological University, UNITED STATES

## Abstract

**Background:**

When confronted with a stressor, animals react with several physiological and behavioral responses. Although sustained or repeated stress can result in severe deleterious physiological effects, the causes of stress in free-ranging animals are yet poorly documented. In our study, we aimed at identifying the main factors affecting stress levels in free-ranging wolves (*Canis lupus*).

**Methodology/Principal Findings:**

We used fecal cortisol metabolites (FCM) as an index of stress, after validating the method for its application in wolves. We analyzed a total of 450 fecal samples from eleven wolf packs belonging to three protected populations, in Italy (Abruzzo), France (Mercantour), and the United States (Yellowstone). We collected samples during two consecutive winters in each study area. We found no relationship between FCM concentrations and age, sex or social status of individuals. At the group level, our results suggest that breeding pair permanency and the loss of pack members through processes different from dispersal may importantly impact stress levels in wolves. We measured higher FCM levels in comparatively small packs living in sympatry with a population of free-ranging dogs. Lastly, our results indicate that FCM concentrations are associated with endoparasitic infections of individuals.

**Conclusions/Significance:**

In social mammals sharing strong bonds among group members, the death of one or several members of the group most likely induces important stress in the remainder of the social unit. The potential impact of social and territorial stability on stress levels should be further investigated in free-ranging populations, especially in highly social and in territorial species. As persistent or repeated stressors may facilitate or induce pathologies and physiological alterations that can affect survival and fitness, we advocate considering the potential impact of anthropogenic causes of stress in management and conservation programs regarding wolves and other wildlife.

## Introduction

When confronted with a physical or psychological stressor that threatens their integrity [1,2], animals react with numerous physiological and behavioral responses to cope with the situation and reestablish homeostasis [3,4]. The glucocorticoids (GCs) cortisol and corticosterone are front-line stress hormones [5]. While adaptive in the short-term, chronic elevated GCs levels consecutive to a prolonged or repeated exposure to a stressor become deleterious [2,6–8]. Besides an immunosuppressive effect favoring pathogenic infections, chronic high cortisol concentrations can cause neuronal cell death, muscle and bone atrophy, poor wound healing, as well as inhibition of growth and reproduction [1,6,8,9]. Even though exposure to persistent stress is therefore a weakening factor that can severely impair health and survival [2,8,10–12], and may impact population dynamics through reduced resistance to diseases [13,14], the study of stress in free-ranging populations is still an emerging field of research [1,15].

Although the negative impact of sustained or repeatedly elevated GCs levels has mainly been described in captive animals, "routes of fecal steroid excretion, general reproductive parameters (…), and general metabolic functions, can reasonably be expected to be very similar, if not the same", in wild and captive animals [16]. In accordance with this, previous studies of free-ranging social mammals and birds report a negative relationship between GCs levels and fitness [10–12,14,17], though the direction of this relationship appears globally inconsistent across species [18]. Whereas a chronic stress response caused by high predation risk may be adaptive in prey species with intermediate life spans [19], we are not aware of previous data suggesting an adaptive value of chronic or repeated stress in long-lived highly social predators such as wolves (*Canis lupus*). Recently developed non-invasive techniques enable the measurement of glucocorticoid metabolites in feces [2,20,21], allowing large-scale and repeated investigations in free-ranging animals without affecting their behavior or physiology. Cortisol is the main secreted glucocorticoid in numerous mammals [13], and fecal cortisol metabolites (FCM) are successfully used as an index of stress in several species [15], including the dog (*Canis lupus familiaris*) [22].

Although animals use various behavioral strategies to decrease or even elude exposure to stressors, these are not always avoidable [23,24]. Environmental stressors identified in different mammals include anthropogenic disturbances [18,25–28], extreme temperatures [1,2], elevated population density [29–31], food limitation and infection by pathogens [8,32]. At the individual level, GCs secretion can vary with age, sex, reproductive status or body condition [1,2,13]. Variations dependent on the temperament or personality of individuals were also suggested [1,33–36]. In social species, interactions with group members can also affect stress levels [37,38], while the relationship with social status remains variable or uncertain [7,29,34]. At the group level, an increase of FCM levels with group size was reported in lions (*Panthera leo*) [39], while the effect of this factor varies with sex in ring-tailed lemurs (*Lemur catta*) [40]. Low stress may correlate with the stability of the social environment [7,38]. In particular, sudden changes such as the loss of an attachment figure, e.g. the mother or the mate in pair-bonding species [41,42], or the disruption of a strong relationship with a member of the group [43] are reported as important stressors. However, the impact of global social instability on established groups remains largely unknown in free-ranging social species. Indeed, most studies of the causes of stress in social species focused on the impact of aggressive interactions and of factors related to dominance hierarchies [29].

Wolves are highly social and typically live in family-based packs consisting of a mated pair and their offspring of the past 1 to 4.5 years [44,45]. In a stable pack, dominance relationships are typically age-graded, with the parents being naturally the top ranking (hereafter 'dominant') individuals, and the only breeding members of the pack [46]. Multiple litters within a group may however occur, particularly when one or both of the original breeders are lost, or in newly founded packs [47–50]. When former breeders become no longer reproductively active before their death, they may keep taking part in all other activities of the group [46]. Mating season takes place once a year during late winter. Most males and females usually reach sexual maturity as yearlings at about 22 months of age [51,52]. Despite all sexually mature females of a pack having a normal estrous cycle and ovulating [46,51,53], wolves are essentially monogamous and the breeding partners of established packs often pair for a lifetime [46,47]. After 61–64 days of gestation [52], an average of 4–6 pups are born in April-May, which greatly benefit from extended alloparental care by the pack members of both sexes [44,46]. While growing up in the pack, the offspring learn social communication, hunting and foraging strategies, and gradually take part in the rearing of pups and territorial defense [44,46]. Most wolves disperse from their natal pack between 9 and 36 months of age [46], alone or in groups, with little known consistent difference between males and females in dispersal characteristics [44]. Dispersers may be accepted in existing packs, in particular after the loss of a breeding partner [44,45,54]. Although packs appear socially less cohesive in summer compared to winter [55], the litter of pups is the social core of the group [44,56] and the social status of dominant individuals does not change throughout the year. Pack members may repeatedly leave and return to their natal pack at any time of the year, before ultimately dispersing. Dispersal most often takes place in fall, or from mid-winter to early spring, i.e. between the mating and the denning seasons [44].

Sex differences in stress levels, as measured from fecal samples [57] or urine samples [58], were not found in wolves. Whereas higher GCs levels were reported in identified [57] or suspected [59] dominant compared to subordinate free-ranging wolves, no correlation between social status and GCs was found in a captive pack [58]. No conclusive link between stress levels and within-pack agonistic encounters could be concluded from these studies, but they reported an increase of GCs during the annual mating season [57], at least in some individuals [58]. To our knowledge, social or territorial stability of packs was never investigated as a potential stressor in wolves. Since direct confrontations with neighboring packs can be severely injurious [44,46], stress levels might depend on the density of the population at a regional scale [31], pack size, and the overall duration of extra-territorial travels. Wolves are well adapted to winter conditions [60], and previous investigations found no relationship between GCs levels and snow pack or minimum temperature [57]. In addition, no seasonal or circadian variation in GCs concentrations has been found in the species [53,58]. Snowmobile activity was associated with elevated FCM levels in wolves [27], and is the only anthropogenic stressor investigated in free-ranging populations that we are aware of.

In this study, we measured FCM levels in fecal samples collected from eleven wolf packs belonging to three protected populations in Europe and North America. We tested for the effect of intrinsic and environmental factors on FCM levels at the individual, group and population levels. As intrinsic factors, we (i) tested for the effect of age, sex, and social status, and combinations including monthly variations in relation to reproductive status and mating season, and (ii) investigated the possible impact of pack social and territorial stability. To explore the impact of putative environmental stressors, we (iii) tested for variations in FCM levels in relation with wolf density, pack size, the presence of free-ranging dogs, and month, and (iv) investigated the possible relationship between FCM levels and endoparasitic infections.

## Material and Methods

### Study areas

We considered wolf packs from three geographical areas: Abruzzo, Lazio e Molise National Park (hereafter: Abruzzo) in central Italy, Mercantour National Park (Mercantour) in south-eastern France, and the northern range of Yellowstone National Park [60] (Yellowstone) in north-western United-States (Table 1). The three study areas are located at similar latitudes, share a mountainous backcountry, a mosaic of forested and open habitats, and are exposed to comparable seasonal climatic variations. At the time of sample collection, wolf packs had settled in all three national parks for at least 20 years.

**Table 1:** Characteristics of the three study areas.

Study area	National park establishment year	Location and coordinates	Mountain range	Wolf presence / return ^a^	Wild ungulates	Livestock	Tourism
**Abruzzo**	1923	Central Italy (41°76' N; 13°84' E)	Apennines	Always present	Chamois ^b^, roe deer, red deer and wild boar	Sheep, horses, cattle, few goats	Important, mostly in summer
**Mercantour**	1979	South-eastern France (44°18' N; 7°05' E)	Alps	1992	Chamois, European mouflon, roe deer, red deer, wild boar, ibex	Sheep, few goats and cattle	Important, mostly in summer
**Yellowstone**	1872	North-western USA (44°60' N; 110°55' O)	Rocky Mountains	1995	Red deer, bison, mule deer, white-tailed deer, moose, pronghorn antelope, big horn sheep, mountain goat	None	Important, mostly in summer

Abruzzo: Abruzzo, Lazio e Molise National Park; Mercantour: Mercantour National Park; Yellowstone: Yellowstone National Park; ind.: individuals.

^a^ Abruzzo: [61]; Mercantour: [62]; Yellowstone: [63].

^b^ Chamois (*Rupicapra rupicapra*), roe deer (*Capreolus capreolus*), red deer (*Cervus elaphus*), wild boar (*Sus scrofa*), european mouflon (Ovis orientalis), alpine ibex (*Capra ibex*), bison (*Bison bison*), mule deer (*Odocoileus hemionus*), white-tailed deer (*Odocoileus virginianus*), moose (*Alces alces*), pronghorn antelope (*Antilocapra americana*), big horn sheep (*Ovis canadensis*), and mountain goat (*Oreamnos americanus*).

The study areas differ in the anthropogenic use of the landscape. Non-existent in Yellowstone, pastoralism is important in Abruzzo and Mercantour, where livestock is present year round in some areas and is part of the diet of wolves. Tourism is a significant activity in all three national parks, but is most important in summertime. Snowmobiling is forbidden in all three areas. Backcountry skiing and snowshoeing are anecdotal in Abruzzo and in Yellowstone, while more common in Mercantour. In Abruzzo, a tiny ski resort running since the 1970's attracts only few visitors, and occupies a very restricted portion of the Iorio pack's territory. Across winters of sampling, there was no known change in human activities within each of the three parks; prey base of wolves was not limiting, and no wolf was legally destroyed in the investigated populations (Table 2).

**Table 2:** Ecological characteristics of the investigated wolf populations at the time of sample collection.

Study area	Wolf density ^a^ (ind./ 1000km)	Main prey species ^b^	Prey base ^c^	Logging and hunting activities in part of the territory of the studied packs ^d^	Recovered dead wolves during years of study (p/c/n/u) ^e^	Dogs ^f^	Density / status of free-ranging dogs ^g^
**Abruzzo**	50	Varied: mainly wild boar, but also roe deer, red deer and domestic ungulates	Not limiting	Yes	10/1/4/3	Pet dogs, working dogs, and stray and feral dogs	High / tolerated, roaming as single or in groups
**Mercantour**	11.5	Varied: mainly chamois and roe deer, but also red deer, ibex, European mouflon, wild boar, and few domestic ungulates (sheep and goats)	Not limiting	Yes	1/2/0/0	Pet dogs and working dogs	Very low / prohibited (controlled)
**Yellowstone**	50	Specific: ≥ 96% red deer; few bison, mule deer, white-tailed deer, and moose	Not limiting	Negligible	0/2/32/4	Pet dogs	Inexistent / prohibited (controlled)

Abruzzo: Abruzzo, Lazio e Molise National Park; Mercantour: Mercantour National Park; Yellowstone: Yellowstone National Park; ind.: individuals.

^a^ Abruzzo: mean estimated value [66]; Mercantour: calculated as the mean number of wolves per pack divided by the mean estimated size of packs' territory in the park (estimated territory size: 260–350km^2^, [67,68]); Yellowstone: information for the northern range of the park [69].

^b^ Wolf fecal samples collected in Abruzzo and Mercantour were submitted to dietary analyses (Abruzzo: P. Ciucci and collaborators, [66]; Mercantour: C. Duchamp and collaborators, [70,71]). In Yellowstone, main prey species were assessed through close monitoring of packs [48–50,67].

^c^ Abruzzo: [60]; Mercantour: Millischer pers. comm.; Yellowstone: [72].

^d^ Abruzzo: [73]; Mercantour: Millischer pers. comm.; Yellowstone: [74].

^e^ Wolves recovered in the study areas and adjacent areas expected to belong to the territory of wolf packs resident of the park. Cause of death: poaching / collision / natural (intraspecific strife)/ unknown. Abruzzo: 2006–2008 (Gentile pers. comm.); Mercantour: 2005–2007 (Millischer pers. comm.); Yellowstone: 2007–2009 (Smith pers. comm.). Five of the 32 individuals who died from natural causes in Yellowstone were members of the studied packs. No such information is available for the two other study areas.

Note: No wolf was legally destroyed in Mercantour or in Abruzzo in the years of sample collection. In Yellowstone, wolf hunting and trapping season first opened in September 2009, a few months after our sample collection was finished; four members of resident packs where legally shot before the end of the year.

^f^ Dogs (*Canis lupus familiaris*) travelling with tourists are prohibited in Abruzzo, allowed in the buffer zone but excluded from the core area of Mercantour, and restricted to a range of 100 yards off roads and parking lots in Yellowstone. Working dogs are shepherd dogs and livestock-guarding dogs.

^g^ Abruzzo: [64,65]; Mercantour (Millischer pers. comm.); Yellowstone: [75].

The three areas differ by the density of wolves and of free-ranging dogs. While stray and feral dogs are absent from Yellowstone and very rare in Mercantour, an important free-ranging dog population lives sympatrically with wolves in Abruzzo, and often relies on the same food sources as wolves [64,65] (Table 2). Mating season in wolves takes place mainly in February in Yellowstone (Molnar pers. obs.), about late February and early March in Abruzzo (Ciucci pers. comm.), and around mid-March in Mercantour (Millischer pers. comm.).

### Investigated packs

We studied a total of eleven wolf packs, and considered two consecutive winters in each investigated national park (Table 3). We defined a pack as a minimum of one male and one female travelling together. This criterion was effective both winters in each group, as indicated by direct observation in Yellowstone, genetic analyses in Mercantour (Duchamp pers. comm.), and confirmed by pup production in Abruzzo [66]. In both Abruzzo and Mercantour, we focused our study on four packs, which we selected based on the quality and quantity of collected fecal samples. Snow-tracking sessions in wintertime, complemented in Mercantour by genetic analyses [67], provided information on pack size (Table 3). In Yellowstone, we studied three different packs, selected based on the visibility of individuals and the accessibility of sample collection sites. To locate the packs, we relied on tracks, howls and bird activity near carcasses. We also collaborated with the local crew, who used telemetry. We monitored the packs daily, from dawn to dusk, whenever weather conditions and distance to the animals (approximately 100 to 1500 meters) allowed sufficiently detailed observation [76].

**Table 3:** Group size and number of fecal samples collected from the investigated wolf packs in the three study areas, 2005–2009.

Study area	Winter of sample collection	Packs	Number of individuals/pack ^a^	Number of collected fecal samples
**Abruzzo**	**2006–2007**	Iorio	6	13
		Orsara	3	19
		Villavalelonga	7	20
		Mainarde	9	27
		**Total**		**79**
	**2007–2008**	Iorio	4	10
		Orsara	6	58
		Villavalelonga	6	6
		Mainarde	5	12
		**Total**		**86**
**Mercantour**	**2005–2006**	Haute Tinée	3–4	18
		Moyenne Tinée	2–3	12
		Vésubie-Roya	3–5	10
		Vésubie-Tinée	3–5	21
		**Total**		**61**
	**2006–2007**	Haute Tinée	2–4	22
		Moyenne Tinée	2	3
		Vésubie-Roya	4–5	13
		Vésubie-Tinée	3–5	22
		**Total**		**60**
**Yellowstone**	**2007–2008**	Slough Creek	14	28
		Druid Peak	16	71
		Blacktail Deer Plateau	(not constituted yet)	-
		**Total**		**99**
	**2008–2009**	Slough Creek	(disappeared) ^b^	-
		Druid Peak	16	34
		Blacktail Deer Plateau	7–10	31
		**Total**		**65**

Abruzzo: Abruzzo, Lazio e Molise National Park; Mercantour: Mercantour National Park; Yellowstone: Yellowstone National Park.

^a^ Abruzzo: based on snow-tracking sessions [66]; Mercantour: based on snow-tracking sessions and genetic analyses performed on fecal samples [67,68,77]; Yellowstonebased on direct observations; variations are due to dispersal and death.

^b^ All pups born in the spring 2008 died, likely in a disease outbreak [49,78]. In summer and fall 2008, three yearlings and three adults died, partly in confrontations with neighboring groups and partly possibly from infectious disease [49]. The group was not found in the winter 2008–2009.

### Sample collection

In Abruzzo and Mercantour, we were given access to fecal samples collected year-round by local scientists and rangers for the purpose of different projects [66,67]. We retained the samples collected between October 1^st^ and March 31^st^, from fall 2005 to spring 2007 in Mercantour and from fall 2006 to spring 2008 in Abruzzo. In Yellowstone, we collected samples from December 1^st^ to March 31^st^, from fall 2007 to spring 2009 (Table 3).

In Abruzzo and Mercantour, most samples were collected within 24 to 48 hours following snowfalls while snow-tracking the studied packs, thus directly identifying the contributing group [66,67]. In Abruzzo, multiple criteria were used to conservatively discriminate wolf scats from those of free-ranging dogs and foxes (*Vulpes vulpes*), among which scat size and absence of tracks from other canids [66,79]. Additional criteria helped discriminate wolf packs from dog groups: variability in step length and foot size between individuals, degree of overlapping among tracks of different individuals, and differences in trajectories in terms of consistency of direction (Ciucci pers. comm.). Based on mtDNA and nuclear markers [80], all fresh scats (n = 107) collected in Abruzzo from December 2005 to March 2006 were from wolves, except two samples from foxes. This provided a direct validation of the selection criteria adopted in this study area (98% accuracy). In Mercantour, systematic genetic analyses are performed on the samples to discriminate wolf scats from those of other species [81]. In both Abruzzo and Mercantour, in absence of snow, samples were collected at known scent posts or exploited carcasses, or during opportunistic surveys along trails [66,67].

In Yellowstone, we collected fecal samples following direct observation and filming of contributing individuals. Daily monitoring allowed the selection of only those samples that stayed at or below freezing point from defecation to collection time. When an individual was observed defecating, we filmed the animal and the surrounding landscape in details (Canon XL-H1 camcorder, Canon EF Adapter XL, Canon EF 100–400mm f/4.5–5.6L IS USM photo lens, Canon Extender EF 2x II). On the same day, we visualized the recorded sequence on a computer and took relevant pictures of the computer screen with a digital still camera. The next day, or as soon as possible in accordance with park regulations, we collected fecal samples using the following procedure: We set a spotting scope at the exact place from where we filmed the defecating individual. From this place, one person guided a collaborator to the samples using walkie-talkies, with the help of the pictures taken with the still camera. During these field trips, when no tracks of other canid species were found less than one meter away from the samples, we collected all scat found on wolf tracks. We abandoned sample collection when one or several wolves not belonging to the target pack were known to have used the area between scat production and collection time. This collection procedure provided information on the age, sex, social status and identity of a number of contributing individuals.

In all three study areas, we discarded fecal samples partly consumed by birds, since only homogenized intact samples can be considered due to the pulsatile secretion of glucocorticoids in response to a stressor [2,82]. As environmental factors can modify the concentrations of FCM levels [13], we also excluded samples that were dried out, exposed to rain or to temperatures obviously above freezing point. We additionally discarded scats over-marked with urine or less than 50 cm away from another scat. Finally, we excluded samples mostly composed of hair (estimated as > 90% of the scat volume) when fecal material content was not sufficient for analysis. On the day of collection, all samples were stored at -20°C in labeled plastic bags and kept frozen until analysis.

### Measurement of fecal cortisol metabolites (FCM)

We first removed any adherent snow from fecal samples. As soon as thawed enough, we vigorously hand-mixed each sample for 1 min through its plastic bag container, in order to homogenize the distribution of FCM in the sample [2,82]. We then suspended a 0.50 ± 0.01g portion of each sample in 5 ml of methanol (80%) to extract cortisol metabolites [83]. After thorough shaking for 30 min, followed by centrifugation at 2500 x g for 15 min, the supernatant was removed and stored at -20°C until further analysis. An aliquot was then processed by a cortisol enzyme immunoassay (EIA). Details of the EIA including cross-reactivity of the antibody are given in Palme and Möstl [84]. Assay sensitivity was 0.3 ng/g, and intra- and inter-assay coefficients of variation were 8.9% and 11.1%, respectively. Each sample was run in duplicate and results expressed as ng/g wet feces. When difference between the two measures exceeded 10%, the sample was reanalyzed. We analyzed all samples in a single laboratory.

The measurement of glucocorticoid metabolites in feces and droppings is a well-established technique [2,15,20,21,84] and the cortisol EIA was successfully validated for dogs [22,85]. Although wolves and dogs belong to the same species [86] and are thus genetically very close [87], we performed a biological validation to confirm the suitability of the EIA for wolf fecal samples. We tested three captive individuals (two females and one male) housed at the 'Popoli Wolf Sanctuary', Italy. As these animals were not accustomed to handling by humans, we used capture (on day 0) for veterinarian control purposes as a biological stressor [2,82]. From day -3 through day +5, fecal samples of each of the three individuals were collected within minutes after defecation and immediately stored in labeled plastic bags at -20°C until analysis.

### Pack stability and composition

In Yellowstone, detailed knowledge of the behavioral dynamics of the studied packs [48–50,76,88] provided information on territorial and social stability of groups. We coded pack territorial stability during the winters of sample collection as a dummy variable, where 1 = stable and 0 = unstable. To investigate pack social stability, we used mortality and dispersal rates, in a period of 12 months including sample collection, from April to March. This period was restricted to the winter of sample collection for the newly founded Blacktail Deer Plateau pack. The death and dispersed rates were, respectively: in 2007–2008, 0 and 0% for the Druid Peak pack, and 20 and 0% for the Slough Creek pack; in 2008–2009: 0 and 27% for the Druid Peak pack, and 10 and 20% for the Blacktail Deer Plateau pack.

In 2007–2008, the Druid Peak pack spent most of its time in the core of its long-established territory [49], and all pack members from 2007 (three adults, six yearlings and seven pups) remained in the group. In November 2008, five yearling males and the long-standing second ranking male (M302) dispersed together [49]. From then on that winter, the pack (eight adults, two yearlings and six pups) repeatedly undertook extensive extra-territorial travels [48,49].

The Slough Creek pack (winter 2007–2008; Table 3) repeatedly experienced severe social instability. In summer 2007, three pack members were killed in intraspecific encounters, and the breeding male died in September, hit by a car. A disperser from a neighboring pack filled the vacant breeding position. No pup survived in 2006 [88], because of the siege of the pack's den site by an unknown pack at the onset of the denning period. These events left the pack with seven adults and nine pups but no yearling, by the end of year 2007.

Finally, the newly established Blacktail Deer Plateau pack (winter 2008–2009, Table 3) was founded in November 2008 by six male and four female dispersers, respectively from the Druid Peak pack and the Agate Creek pack [49]. The group travelled extensively before settling in a new territory. Three founders went missing during the winter: two yearling males dispersed, while a yearling female was most likely killed late November in a confrontation with the Druid Peak pack [49,50], leaving the group with three adults (one male, two females) and four yearlings (three males, one female).

### Parasitological data

We identified helminth eggs and protozoan cysts through microscopic examination in 303 of the fecal samples. As soon as thawed enough, we vigorously hand-mixed fecal samples for 1 min through the plastic bag. We then placed 1.55 ± 0.05 g of feces in a plastic container, mixed with 10 ml of sodium acetate—acetic acid—formaldehyde (SAF) fixative [89] and kept the suspension at 4°C until it was tested. To detect protozoan cysts, helminth eggs and larvae, we used the following modified SAF concentration technique [89]: We filtered the fecal suspension through a strainer and centrifuged a 4 ml aliquot with 5 ml of physiologic solution (NaCl 0.85%) for 10 min at 500 x g. We removed the supernatant and resuspended the pellet in 6 ml of physiologic solution, and added 3 ml of ether. We vortexed the solution for 30 seconds, and then centrifuged it for 10 min at 500 x g. After removing the supernatant, we dissolved the pellet in a few drops of saline allowing adequate observation under the microscope. We prepared both stained and unstained preparations for each sample [90]. To obtain a stained preparation, we added a drop of 5 x diluted Lugol solution [91] to a drop of the concentrated fecal solution deposited on a slide. To observe unstained structures, we added a drop of saline to the concentrated fecal solution. After mixing with the corner of a coverslip (18x18 mm), the latter was used to cover the preparation. We examined each preparation by systematically scanning the entire coverslip at 100x magnification, using a calibrated Olympus BX50 microscope, and used 400x magnification to confirm each observation. We identified parasite eggs on the basis of their size, color, shape, look of content, and structure of the shell surface. Eggs and cysts were identified to genus or species level [92–102], except for the Taeniidae family whose eggs cannot be discriminated by microscopic examination [93,96]. Nematode larvae could not be identified and were not considered in the count of detected parasite taxa. We defined parasite richness as the number of different parasite taxa detected in a fecal sample.

### Statistical analyses

We tested the effects of covariates on FCM levels using mixed-effects regressions [103] in an information theoretic framework [104]. We built a set of candidate models to explore the effect of the independent variables. We selected for the most parsimonious model based on the AICc model selection criterion corrected for small sample size [104]. When candidate models were within ΔAICc < 2, we estimated unbiased fixed-effects coefficients using model averaging [104, 105]. Averaged-coefficients in the final model were deemed significant when the corresponding 95% confidence interval (CI) did not include zero [104].

For the two packs from which these data were available, we modeled FCM levels as a function of age-class (pup: < one year old; yearling: one to two years old; adult: > two years old), sex, social status (highest ranking male and female, or subordinate), month of collection, and their interactions as fixed-effects. We fitted a random intercept for individual identity nested in pack identity to control for pseudo-replication, and a random intercept for winter of collection to account for unmeasured yearly variation in environmental conditions.

To test for the effect of pack stability on FCM levels, we modeled FCM levels measured in samples from Yellowstone as a function of mortality rate, dispersal rate and month of sample collection. We fitted a random intercept for pack identity to control for pseudo-replication, and for winter of collection.

Using our entire data set, we assessed the impact of potential environmental stressors on FCM levels. We used population density, pack size, presence of free-ranging dogs, and month of sample collection as fixed-effects. We did not include mortality rates in these analyses, as the monitoring of packs in Yellowstone is much more intensive than in the European national parks. Except for Yellowstone, we therefore could not attribute recovered wolf carcasses to specific wolf packs. We modeled interaction effects between presence of dogs and pack size, density and pack size, and density and month. We fitted a random intercept for pack identity nested in study area to control for pseudo-replication, and a random intercept for winter of sample collection. As we were interested in identifying fixed-factors with the strongest effect, we used model averaging with shrinkage in both of these candidate model sets [105].

Lastly, to assess the relation between stress and parasitic infection, we tested for a correlation between FCM levels and parasite richness using a Spearman’s rho correlation across the entire data set, and separately within each population.

We ran all statistical tests in R version 3.1.2 [106]. We fitted mixed-models using the package *lme4* (version 1.1–7) [103]. We performed model averaging using the *MuMIn* package (version 1.12.1) [107]. We report mean ± standard error (SE).

### Ethics statement

In Abruzzo, Lazio e Molise National Park, research was approved by the national park authority (Determinazione no. 38 dated 24 March 2003). No specific permission was required for the collection of fecal samples in Mercantour National Park. In Yellowstone National Park, the data were collected in agreement with the park's policy; permits YELL-2007-SCI 5716, YELL-2008-SCI 5716, and YELL-2009-SCI 5716 were delivered by the authority of the national park.

The wolf is protected in all three study areas (Abruzzo, Mercantour and Yellowstone). As the collection of fecal sample is a non-invasive procedure, our study did not require approval by animal ethics committees.

## Results

In our biological validation experiment, measured FCM concentrations ranged from 7.1 to 361.6 ng/g feces. Baseline values, calculated for each tested individual as the mean of the FCM levels measured prior to capture, ranged from 24.0 to 48.4 ng/g feces (when several sample were collected from an individual on the same day, the mean daily FCM level was considered in these calculations). In the three individuals, the cortisol EIA detected an increase in FCM concentrations by 440%, 747% and 1041% respectively (absolute peak values between 198.4 and 361.6 ng/g), within 24 to 48 hours of the capture event. Within 48 to 96 hours, this peak was followed by a downward trend towards baseline FCM values, validating the cortisol EIA for the analysis of wolf fecal samples.

We analyzed a total of 450 samples from Abruzzo (n = 165), Mercantour (n = 121) and Yellowstone (n = 164). Measured FCM ranged from 1.1 to 397.7 ng/g feces. We defined extreme high values as those beyond the limit of Q3 + 3 x IQ, where Q3 = upper quartile, IQ = interquartile range. Hereafter, these values (> 47.2 ng/g feces) are referred to as 'extreme values'. The limit for extreme low values, Q1–3 x IQ (where Q1 = lower quartile) was zero.

### Age, sex and social status

**Fig 1:**
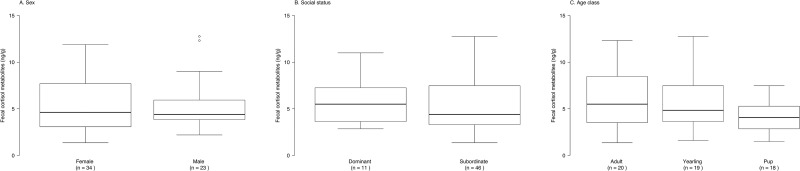
Concentrations of fecal cortisol metabolites in two wolf packs from Yellowstone National Park (USA), 2007–2009, grouped by (a) sex, (b) social status, and (c) age-class. Box and whisker plots show median (horizontal line within box), 25% and 75% percentiles (box), range (whiskers) and statistical outliers (open circles). For clarity, FCM values above 15 ng/g are not shown.

In Yellowstone, we had a complete set of information on the identity, age, sex, and social status of the contributing individuals for 57 fecal samples collected in two packs (Druid Peak and Blacktail Deer Plateau) over two winters. Fecal cortisol metabolites levels were: 7.0 ± 2.3 ng/g (n = 18) in pups, 6.5 ± 1.2 ng/g (n = 19) in yearlings, and 8.3 ± 1.6 ng/g (n = 20) in adults; 6.9 ± 1.7 ng/g (n = 23) in males and 7.6 ± 1.7 ng/g (n = 34) in females; 5.7 ± 0.8 ng/g (n = 11) in dominant individuals and 7.7 ± 1.5 ng/g (n = 46) in subordinate individuals (Fig 1). A reduced model with only an intercept term ranked as the most parsimonious model (Table 4).

**Table 4:** Results of selection of mixed-models exploring the effect of sex, age, social status, and month on fecal cortisol metabolites levels in two wolf packs from Yellowstone National Park (USA), 2007–2009. Only candidate models with ΔAICc < 10 are shown.

Fixed-effects parameters ^a^	k	log likelihood	AICc	ΔAICc	w
**null**	**5**	**-206.1**	**423.3**	**0**	**0.45**
social status	6	-205.9	425.4	2.10	0.16
sex	6	-206.0	425.8	2.43	0.13
month	8	-204.1	427.1	3.80	0.07
sex + social status	7	-205.9	428.0	4.65	0.04
age	7	-205.9	428.1	4.73	0.04
age + social status	8	-204.9	428.8	5.49	0.03
month + social status	9	-203.8	429.5	6.12	0.02
month + sex	9	-204.1	429.9	6.60	0.02
sex + social status + sex x social status	8	-205.8	430.6	7.27	0.01
age + sex	8	-205.8	430.7	7.35	0.01
age + sex + social status	9	-204.9	431.6	8.31	0.01
month + sex + social status	10	-203.8	432.4	9.02	0.00
age + month	10	-204.0	432.7	9.36	0.00

k: number of estimable parameters; AICc: Akaike Information Criteria adjusted for small sample sizes; ΔAICc = (AICc)–(AICc)_min_; w: Akaike weight.

^a^ All models were fitted with a random intercept for individual identity nested within pack identity, and a random intercept for winter of sample collection.

### Pack stability

**Fig 2:**
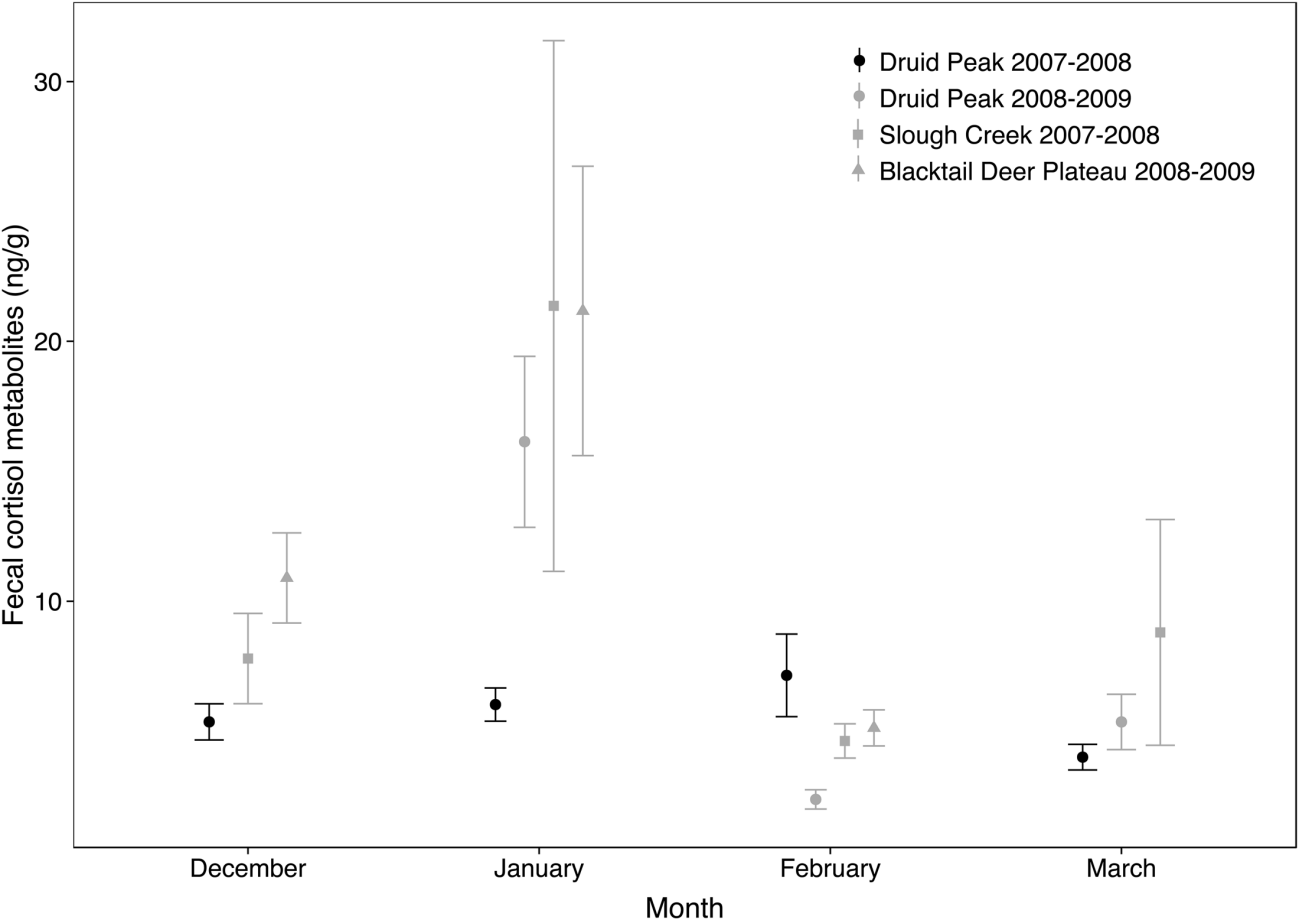
Monthly mean fecal cortisol metabolites levels measured in a socially and territorially stable wolf pack (Druid Peak 2007–2008) and in three unstable packs (Slough Creek 2007–2008, Druid Peak 2008–2009 and Blacktail Deer Plateau 2008–2009) in Yellowstone National Park (USA). Error bars represent standard errors.

In our data set from Yellowstone (n = 164), mortality and month of collection affected FCM levels (Table 5). Packs with higher mortality rates had significantly higher FCM levels (β_mortality rate_ = 29.1, 95% CI 5.5–52.8). Territorial stability and dispersal rate had no significant effect on FCM levels of group members (β_territorial instability_ = 1.1, 95% CI -2.4–4.6; β_dispersal rate_ = 3.7, 95% CI -9.8 –-17.3). Mean FCM levels in January were significantly higher than in the other three months of sample collection (β_Dec_ = -7.4, 95% CI -11.9 –-2.9; β_Feb_ = -7.6, 95% CI -11.9 –-3.3; β_Mar_ = -8.4, 95% CI -15.4 –-1.5). In the socially and territorially stable Druid Peak pack (2007–2008), monthly differences in FCM levels were small compared to other sets of data (Fig 2).

**Table 5:** Results of selection and averaged fixed-effects coefficients of mixed-models exploring the effect of territorial stability, mortality rate, dispersal rate, and month on fecal cortisol metabolites levels in three wolf packs from Yellowstone National Park (USA), 2007–2009. Only candidate models with ΔAICc < 10 are shown. We used candidate models with ΔAICc < 2 (bold face) for model coefficient averaging.

**Model parameters** ^**a**^	**k**	**log likelihood**	**AICc**	**ΔAICc**	**w**
**month + mortality rate + territorial stability**	**9**	**-625.3**	**1269.7**	**0**	**0.26**
**month + dispersal rate + mortality rate**	**9**	**-625.4**	**1270.0**	**0.27**	**0.23**
**month + mortality rate**	**8**	**-626.6**	**1270.1**	**0.35**	**0.22**
month + dispersal rate + mortality rate + territorial stability	10	-625.2	1271.8	2.14	0.09
month	7	-628.6	1271.9	2.17	0.09
month + territorial stability	8	-628.1	1273.2	3.46	0.05
month + dispersal rate	8	-628.3	1273.6	3.89	0.04
month + dispersal rate + territorial stability	9	-627.5	1274.1	4.40	0.03
dispersal rate + mortality rate	6	-633.2	1279.0	9.32	0.00
mortality rate + territorial stability	6	-633.4	1279.2	9.54	0.00
			**95% confidence interval**		
**Fixed-effect parameters**	**averaged β**	**SE**	**lower**	**upper**	
(intercept)	12.0	1.9	8.3	15.8	
territorial instability ^b^	1.1	1.8	-2.4	4.6	
mortality rate *	29.1	12.0	5.5	52.8	
dispersal rate	3.7	6.9	-9.8	17.3	
December ^c^ *	-7.4	2.3	-11.9	-2.9	
February ^c^ *	-7.6	2.2	-11.9	-3.3	
March ^c^ *	-8.4	3.5	-15.4	-1.5	

k: number of estimable parameters; AICc: Akaike Information Criteria adjusted for small sample sizes; ΔAICc = (AICc)–(AICc)_min_; w: Akaike weight;

* parameter deemed significant as confidence interval excludes zero.

^a^ All models were fitted with a random intercept for pack identity and a random intercept for winter of sample collection.

^b^ Reference category was territorial stability.

^c^ Reference category was January.

### Environmental factors

Across our entire data set (n = 450), FCM levels were positively affected by population density, although this effect was not informative in the final averaged model (β_density_ = 0.03, 95% CI -0.3–0.3; Table 6). The presence of free-ranging dogs and pack size significantly affected FCM levels. There was a significant interaction between the presence or absence of free-ranging dogs and pack size (β_no dogs x pack size_ = 6.7, 95% CI 3.3–10.0), with smaller packs being more stressed than larger packs in the presence of dogs (β_pack size_ = -7.0, 95% CI -10.2 –-3.9). The presence of a population of free-ranging dogs was associated with higher FCM levels (β_no dogs_ = -56.1, 95% CI -81.6 –-30.6), setting the wolf population of Abruzzo aside.

**Table 6:** Results of selection and averaged fixed-effects coefficients of mixed-models exploring the effect of wolf population density, pack size, presence of a sympatric free-ranging dog population, and month of sample collection on fecal cortisol metabolites levels in wolves in Abruzzo, Lazio e Molise National Park (Italy), Mercantour National Park (France), and Yellowstone National Park (USA), 2005–2009. Only candidate models with ΔAICc < 10 are shown. We used candidate models with ΔAICc < 2 (bold face) for model coefficient averaging.

**Fixed-effects parameters** ^**a**^	**k**	**log likelihood**	**AICc**	**ΔAICc**	**w**
**dogs + pack size + dogs x pack size**	**8**	**-2180.0**	**4376.3**	**0**	**0.50**
**density + dogs + pack size + dogs x pack size**	**9**	**-2179.9**	**4378.2**	**1.89**	**0.20**
dogs + pack size + month + dogs x pack size	13	-2176.1	4379.0	2.67	0.13
density + dogs + pack size + density x pack size + dogs x pack size	10	-2179.8	4380.1	3.80	0.08
density + dogs + pack size + month + dogs x pack size	14	-2176.1	4381.1	4.79	0.05
density + dogs + pack size + month + density x pack size + dogs x pack size	15	-2175.8	4382.8	6.51	0.02
density + pack size	7	-2184.7	4383.6	7.28	0.01
density + pack size + density x pack size	8	-2184.6	4385.6	9.32	0.00
density + dogs + pack size	8	-2184.7	4385.6	9.35	0.00
density + dogs + pack size + month + density x month + dogs x pack size	19	-2173.0	4385.7	9.43	0.00
			**95% confidence interval**		
**Fixed-effect parameters**	**averaged β**	**SE**	**lower**	**upper**	
(intercept)	69.1	12.6	44.3	93.8	
density	0.03	0.2	-0.3	0.3	
no dogs ^b^ *	-56.1	13.0	-81.6	-30.6	
pack size *	-7.0	1.6	-10.2	-3.9	
no dogs x pack size *	6.7	1.7	3.3	10.0	

k: number of estimable parameters; AICc: Akaike Information Criteria adjusted for small sample sizes; ΔAICc = (AICc)–(AICc)_min_; w: Akaike weight;

* parameter deemed significant as confidence interval excludes zero.

^a^ All models were fitted with a random intercept for pack identity nested within study area, and a random intercept for winter of sample collection.

^b^ Reference category was free-ranging dog population present.

**Fig 3:**
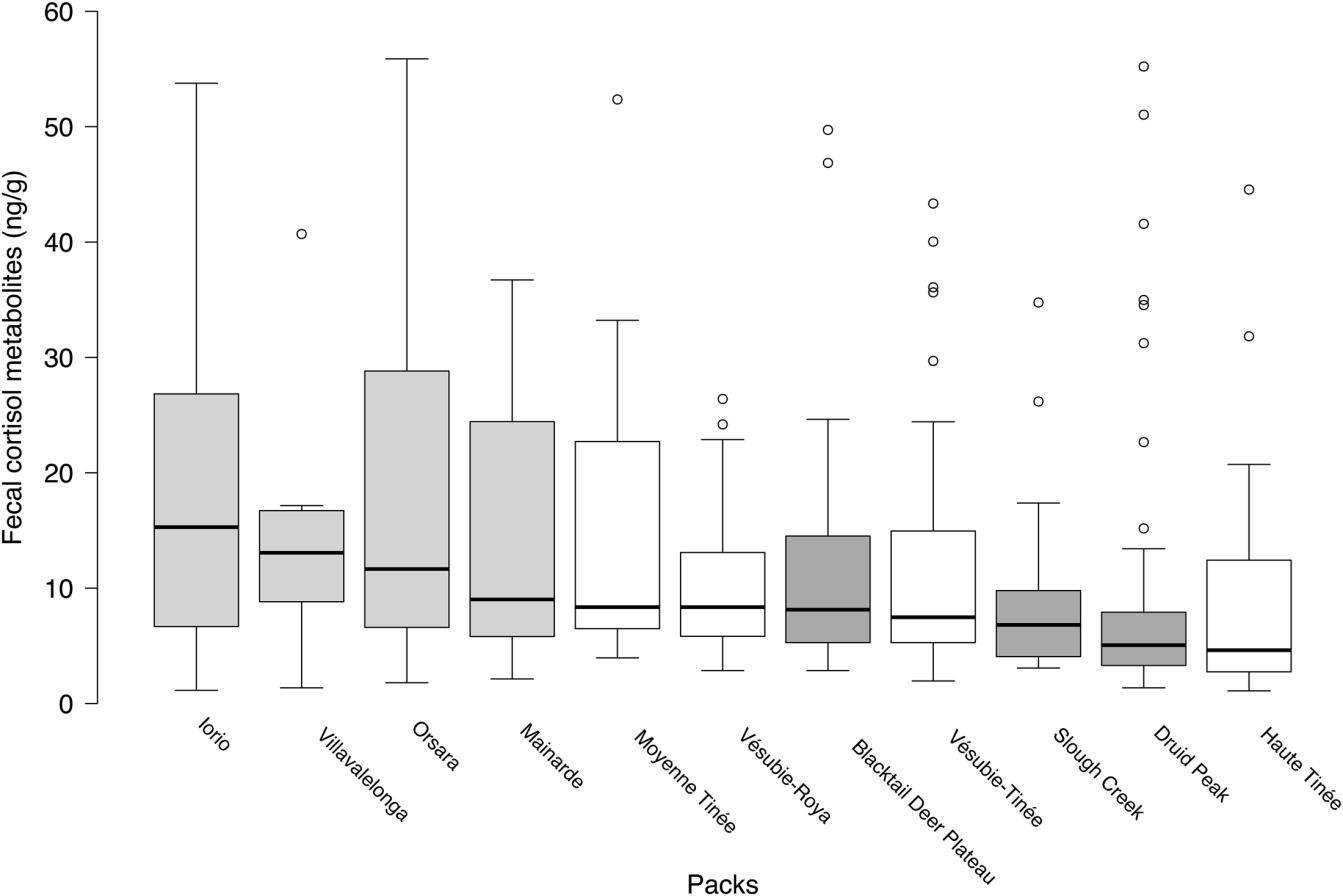
Concentrations of fecal cortisol metabolites (FCM) in 11 free-ranging wolf packs from Abruzzo, Lazio e Molise National Park (2006–2008, n = 165), Italy (light grey), Mercantour National Park (2005–2007, n = 121), France (white), and Yellowstone National Park (2007–2009, n = 164), USA (dark grey). Box and whisker plots show median (horizontal line within box), 25% and 75% percentiles (box), range (whiskers) and statistical outliers (open circles). For clarity, outliers above 60 ng/g are not shown.

Comparison of FCM levels across the eleven packs confirmed clustering of elevated values in Abruzzo (Fig 3). Mean FCM levels were 27.3 ± 3.9 ng/g (n = 165) in Abruzzo, 11.6 ± 1.1 ng/g (n = 121) in Mercantour, and 9.4 ± 0.9 ng/g (n = 164) in Yellowstone. Extreme values of FCM (n = 30) were rare in Mercantour (n = 2, from one pack, in February and March) and in Yellowstone (n = 4, from all packs, in January), while frequent, measured in all packs in both winters, and in most studied months (10/12), in Abruzzo (n = 24).

**Fig 4:**
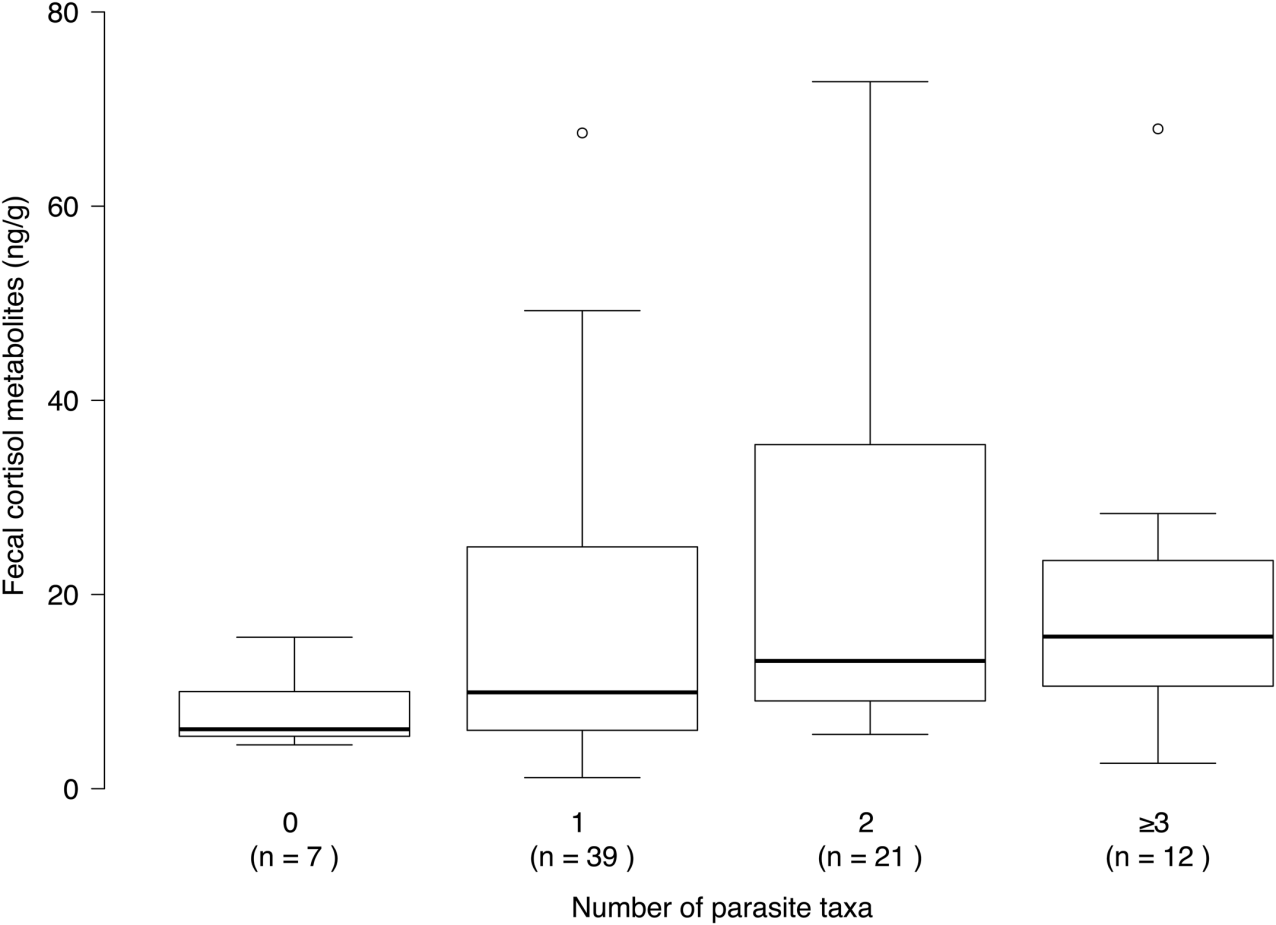
Concentrations of cortisol metabolites and detected number of parasite taxa in wolf fecal samples collected in Abruzzo Lazio e Molise National Park (Italy), from October 2006 to March 2007. Box and whisker plots show median (horizontal line within box), 25% and 75% percentiles (box), range (whiskers) and statistical outliers (open circles). For clarity, outliers above 80 ng/g are not shown.

Considering parasite richness, we examined 164 samples from Yellowstone, collected in both winters, and 79 and 66 samples from the winter 2006–2007 in Abruzzo and in Mercantour respectively. We found three or more parasite taxa per fecal sample in 15.2% of the analyzed samples from Abruzzo, 3.0% from Yellowstone, and 0.0% from Mercantour. In Abruzzo, we found a significant correlation between FCM levels and parasite richness (Fig 4, r_s_ = 0.266, n = 79, p-value = 0.018). We obtained similar results when discarding extreme values (r_s_ = 0.337, n = 66, p-value = 0.006). In Yellowstone and in Mercantour, we found no significant correlation between parasite richness and FCM levels.

## Discussion

We found no relationship between FCM levels and age, sex, or social status in the samples for which the identity of the contributing individuals was known. In Yellowstone, where details on behavioral dynamics of packs was known, our results show that mortality within packs was the only factor of stability affecting FCM levels. Higher FCM levels in January during pre-estrous in female wolves, compared to the other considered months, was also highlighted in these data. Considering our entire data set, we found that larger pack size is associated with lower FCM levels in the presence of a sympatric free-ranging dog population.

We selected the protected wolf populations of Abruzzo, Mercantour and Yellowstone in order to exclude climatic differences [1,2] and legal human-caused mortality that could bias our investigations. We standardized sample selection procedures and validated the cortisol EIA assay for wolf fecal samples. These precautions assured the reliability of the measured FCM concentrations, which therefore adequately reflect adrenocortical activity and stress levels in individuals and packs. Measured FCM concentrations in samples collected during the biological validation experiment and in samples collected in the study areas were within a similar range. We limited sample collection to colder months of the year as the best possible strategy to avoid bias caused by climatic factors in the measurement of FCM levels [13]. These precautions helped ensure consistency of the results and allowed the evaluation of the relationship between selected factors and FCM levels. Since no seasonal or circadian variation in cortisol level has been detected in wolves [53,58], we assumed no impact of the opportunistic collection procedure on our results.

In Abruzzo and Mercantour, the diet of wolves is diverse, consisting of a variety of ungulate species, while wolf packs on the northern range of Yellowstone almost exclusively feed on elk. A detailed dietary analysis conducted on all wolf scats collected in Abruzzo [66] between January 2006 and October 2008 revealed important differences among the four studied packs in the prey species preferentially selected, be it in the winters of our study or during the rest of the year [66]. However, we found no significant difference among these packs in FCM levels globally measured in the data set from Abruzzo. Together, these data suggest that neither the overall diversity of consumed prey species, nor the preferential selection of specific prey species, affected FCM levels in the considered wolf populations. Lastly, if a hypothesized influence of GCs acquired through the consumption of prey [108] was demonstrated, we suggest that it would most likely be similar across our entire data set.

### Age, sex and social status

We considered age, sex, social status, and combinations including monthly variations in relation to reproductive status and mating season, as intrinsic factors potentially acting on stress levels in free-ranging wolves. Our results show no relationship between FCM levels and any of these factors, considered alone or in combination with each other. We are not aware of previous investigations of GCs levels in wolves in relation to age-classes. Our results showing no association between sex and FCM levels are in agreement with previous work in wolves [57,58]. Prior studies in this species failed to find differences in hormonal cycles between reproductive and non-reproductive individuals of the same sex, including between pregnant and non-pregnant females [46,53]. Our results indicate that reproductive status does not likely have a direct influence on FCM levels in wolves. In agreement with this, previous studies found no variation of cortisol concentrations in female dogs relative to their reproductive status, reporting a peak in cortisol levels only during labor and at parturition [109,110]. In our study, a likely elevation of FCM levels linked with parturition was not detectable, as delivery occurred outside the period of sample collection. That we found no difference in FCM levels between dominant and subordinate individuals supports [58] or contradicts [57,59] previous conclusions. From an individual (male M302) that changed status from subordinate in the first winter to dominant in the second winter, we even measured two times lower FCM levels in the new leading position (as subordinate: 10.7 ± 1.7, n = 2; as dominant: 5.1 ± 1.1, n = 2), albeit based on a very small sample size.

Individuals of different age, sex or social status might be exposed to stressors of different nature, which would translate into a similar effect on FCM levels. Alternatively, as reported in numerous species, our results suggest that variations in stress levels may be modulated by other factors related to individual differences, such as the personality or temperament [1,33–36,111], or the strength and/or the quality of the social bonds shared with other group members [41–43]. Such individual factors most likely affected FCM levels across our entire data set.

Although a relatively small sample size could limit inference, our results are congruent with all or parts of previous investigations in wolves. As we controlled for pseudo-replication, we are confident that age, sex and social status had likely no effect on FCM levels in these data. In the absence of information on the identity of defecating individuals for all samples, we relied on the assumption that the lack of effect of these intrinsic factors was also true for our entire data set.

### Pack stability and mating season

High measured FCM levels in socially disrupted packs are in accordance with low stress levels correlated to social environment stability reported in other social mammals [7,38]. Our results suggest that the death of pack members causes important stress in the group, while territorial instability and the loss of pack members through dispersal do not appear to be key stressors in free-ranging wolf packs. Abrupt social changes are reported to be important stressors in several social mammals [41–43,112]. In wolves, the loss of one or both of the dominant individuals creates important perturbations in the group, evidenced by an elevated rate of dissolution of such socially disturbed packs [113]. Adult wolves provide important stability to a pack through their knowledge of the territory, experience, and long-established social bonds. But attachment figures can include individuals of all age-classes, such as siblings, caregivers, and other specific social partners [46]. Our results may also reflect the stressful challenge of founding a new social unit. At first, establishing contact with unknown conspecifics is arduous, as the outcome of such encounters is always uncertain. Time is then needed to adjust to the novel social environment and settle in a suitable territory, in a landscape often occupied by other groups.

In Yellowstone, elevated FCM levels in January match the pre-estrus period in females, characterized by an increased attractiveness to males [51]. Mating competition among pack members at the onset of the breeding season might represent a temporary stressor. However, conversely to the packs in which the dominant pair was only recently established, breeding status was not much disputed in the Druid Peak pack (Molnar, pers. obs.), in accordance with incest avoidance commonly recognized in free-ranging wolves [45,114]. Besides the fact that no group member died, a stable breeding pair specifically characterized this pack throughout our study. Our data suggest that breeding pair permanency might buffer the stressful impact of the mating season. The downward trend in FCM levels measured in February in Yellowstone packs may be related to variations in sexual hormone levels [51,115,116], and likely correlates with the negative retro-control initiated by the peak of circulating cortisol [117] in January.

### Environmental factors

Across our entire data set, the results indicate that pack size, the presence of free-ranging dogs, and the interaction between these two factors significantly affected FCM levels in wolves. Lower stress level measured from larger packs contradicts previous observations in lions [39], and may be true only within a certain range of group size. This result might however reflect the higher competitiveness of larger groups for the defense of territory and related resources in long-settled populations, in which all suitable territories are often occupied. The fact that larger packs are less stressed than smaller ones in the presence of sympatric free-ranging dogs can be interpreted in a similar way. Free-ranging dogs are absent from Yellowstone and extremely rare in Mercantour, whereas they are very common in Abruzzo. Although no official assessment is available for Abruzzo, about 190 free-ranging dogs/100 km^2^ have been estimated in 1999 through a sight-resight survey in the nearby Majella National Park, which has very similar ecological and cultural settings (Ciucci pers. comm.). This important population of free-ranging dogs may have a stressful impact on sympatric wolves, possibly through competition [118] related to the defense of a territory and associated food source. Absent from Abruzzo, the solitary Eurasian lynx (*Lynx lynx*) is not an important competitor of wolves in Mercantour. Indeed, the population of the felid is fragmented in the French Alps and at low density compared to other Alpine regions [119,120]. In Yellowstone, sympatric carnivores active in winter are solitary, as pumas (*Puma concolor*), or mainly prey on species different from the elk most commonly used by wolves, as coyotes (*Canis latrans*).

The fact that the presence of free-ranging dogs was associated with elevate FCM levels in our results clearly sets the wolf population of Abruzzo aside. Comparable landscape, climatic conditions, and sufficient prey base characterize the three study areas, and differences in diet did not appear to affect FCM levels. Nevertheless, we measured substantially higher FCM levels in Abruzzo, where we additionally detected most of the extreme values, recorded in all packs during both winters, and in almost every single month of the study. Together, these results suggest that this specific wolf population was under sustained or repeated stress at the regional scale. Many of the identified environmental factors that could have impacted FCM levels in the wolf population of Abruzzo are shared with Yellowstone, such as wolf density, or with Mercantour, such as a varied diet, and logging, hunting and pastoralist activities in part of the territory of the studied packs. Therefore, the main cause of elevated FCM levels measured in Abruzzo is not likely attributable to these factors. Tourism is important in the three study areas, but is mostly substantially reduced in winter. Pastoralism and increasing human density, but not tourism, were reported as meaningful stressors in spotted hyenas (*Crocuta crocuta*), another large social carnivore [26]. Pastoralism is important in both European national parks, although much reduced in winter. Compared to the studied packs in Yellowstone, which spent about 99% of their time within the park boundaries [74], wolves in Abruzzo and Mercantour are more regularly exposed to localized logging, as well as to hunting of wild ungulates taking place outside the boundaries of the parks [73] (Millischer pers. comm.). Indeed, the two European national parks are settled in a landscape more intensively dominated by human activities. Also, because of their small size, part of the territory of wolf packs lies outside the core area of the parks, potentially leading to an important edge effect [121]. Wolves may temporarily restrict their travels in portions of their territory free of such anthropogenic disturbances, as reported in lions [122].

Our results suggest that the death of pack members is an important stressor in free-ranging wolves. During the years of sample collection, the main identified causes of mortality varied notably between the study areas. Besides the presence of a sympatric dog population, the other known environmental factor specifically characterizing the wolf population in Abruzzo is an elevated rate of poaching.

All wolf carcasses recovered in the three national parks and in adjacent areas are systematically examined to investigate the cause of death. The death rates attributable to some of these causes are probably estimated rather accurately (e.g. collision), while others are underestimated (i.e. natural, poaching) [123]. These biases can be expected to be similar in the studied populations, except for natural death and poaching more likely detected in Yellowstone due to intensive monitoring. Thus, detected natural mortality rates in the three study areas cannot be compared.

In social and territorial species, human-caused mortality is expected to importantly impact social organization and spatial distribution of groups (e.g. effects of culling on group-living European badger, *Meles meles* [124,125]). In species establishing strong bonds with conspecifics, social disruption is reported as an important stressor [41–43,126]. Such impact has also been suggested in wolves, with higher cortisol levels measured in hunted populations compared to undisturbed ones [127]. In Abruzzo, 56% of the wolves recovered dead were victims of poaching, while this proportion was of 33% in Mercantour, and 0% in Yellowstone (Table 2). Taking wolf density into account, the number of poached wolves was ≥ 2.3 times higher in Abruzzo compared to the two other study areas. Social disruption as a consequence of poaching is reported to be a chronic stress condition in the African elephant (*Loxodonta africana*, [126]), a species also living in family-based groups sharing strong bonds among group members. As anthropogenic persecution is expected to cause important and recurrent social instability in free-ranging wolf packs [45], the high poaching levels repeatedly reported inside Abruzzo and within its outer buffer area (Gentile pers. comm.) may be a key factor explaining the elevated FCM levels measured in this population.

Besides the development of stress-induced pathologies, the immunosuppressive effect of elevated FCM levels measured in packs from Abruzzo may facilitate pathogenic infections in this wolf population [1,6,8]. In this population, in which infection by more than two endoparasite taxa was most commonly detected, elevated FCM levels were correlated with parasite richness. While elevated stress levels could predispose individuals to multiple infections, infection itself is a physical stressor that can trigger an increase in FCM levels [1,8]. In a feedback process, increased FCM levels may favor infection by additional pathogens. Stress enhances the development of viral diseases otherwise optimally overcome by the immune system [128], as reported for canine coronaviruses [129]. In wolves, stress and different parasitic infections are also predisposing factors to infection by canine parvovirus type 2 [51]. These viruses were detected in fecal sample analyzed in our study, collected in Abruzzo in the winter 2006–2007, and in Mercantour in the winter 2005–2006. Interesting to note, infection was recorded in twice as many packs in Abruzzo as in Mercantour, for each virus [130].

## Conclusions

Our study is among the rare investigations of the possible causes of stress in free-ranging mammals. At the group level, our results suggest that breeding pair permanency might significantly reduce the stressful impact of the annual mating season. At a wider time interval, the loss of pack members through processes different from dispersal was associated with elevated FCM levels. As social disruption is expected to be particularly stressful in highly social species sharing strong bonds among group members [125,126], the death of wolf pack members most likely acts as an important stressor in the remainder of the social unit. In free-ranging populations, mortality rate and turnover in breeding individuals both increase with human persecution.

Besides the death of pack members, our results suggest that the presence of a sympatric free-ranging dog population likely affects stress in wolves. These two factors may also interact in various ways. A prospective assessment of FCM levels in the three study areas could help specify the relative importance of these stressors, as human persecution of wolves has increased in Mercantour and in Yellowstone since our study. While occurring outside the boundaries of the national parks, legal shooting nearby Mercantour, and the opening of a wolf hunting and trapping season around Yellowstone directly impact wolf packs partly or mostly established in these protected areas (Millischer pers. comm., Smith pers. comm.). The potential impact of social and territorial instability on GC levels should be further investigated in free-ranging populations, especially in highly social and in territorial species. As for other anthropogenic causes of disturbances, comparing areas with intermediate densities of free-ranging dogs would help understand the impact of this specific factor on FCM levels in wolves and in other wildlife. The effect of tourism, logging and hunting activities on stress levels should likewise be studied in wolves and in other wildlife, in populations in which these factors can be precisely measured. Finally, prospective studies in free-ranging animals should also investigate the relationship between FCM levels and infectious diseases, including parasitic infections.

Next to the suffering and trauma caused to animals by invasive anthropogenic disturbances, which add up to natural causes of distress [131], persistent or repeated stressors may facilitate or induce pathologies and physiological alterations that can affect survival and fitness. We advocate acknowledging and considering the potential impact of direct and indirect anthropogenic causes of persistent or recurrent stress, in management and conservation programs of wolves and other wildlife.
